# Clinical Outcomes of Custom Foot Orthoses in Progressive Collapsing Foot Deformity: A Retrospective Cohort Analysis

**DOI:** 10.1002/jfa2.70153

**Published:** 2026-04-26

**Authors:** Kelly Robb, Michael Ryan, Gabriel Moisan

**Affiliations:** ^1^ Department of Kinesiology and Physical Education Faculty of Science Wilfrid Laurier University Waterloo Ontario Canada; ^2^ Kintec Footlabs Inc. Surrey British Columbia Canada; ^3^ School of Mechatronics Systems Engineering Simon Fraser University Burnaby British Columbia Canada; ^4^ Groupe de Recherche sur les Affections Neuromusculosquelettiques (GRAN) Université du Québec à Trois‐Rivières Trois‐Rivières Québec Canada; ^5^ Department of Human Kinetics Université du Québec à Trois‐Rivières Trois‐Rivières Québec Canada

**Keywords:** ankle, foot, musculoskeletal Diseases, orthotic devices, physical examination

## Abstract

**Background:**

Progressive collapsing foot deformity (PCFD) is a painful and function‐limiting condition most commonly affecting middle‐aged and older adults. Although custom foot orthoses (CFOs) are routinely prescribed as a conservative intervention, evidence from large clinical cohorts remains limited. This study aimed to characterise patients with PCFD seeking orthotic care, examine demographic factors associated with pain improvement, evaluate clinical and self‐reported outcomes following CFO use, and explore potential interaction effects of CFOs on clinical improvement across sex.

**Methods:**

A retrospective analysis of clinical records between January 1, 2016 and May 31, 2025 extracted 130,365 appointments across 25 Canadian pedorthic clinics, further extrapolated into 669 PCFD encounters and 12,621 asymptomatic controls. Each assessment included pain ratings, Foot Posture Index (FPI), functional tests, gait analysis, and follow‐up surveys on CFOs wear time, comfort, and recovery.

**Results:**

Results identified that PCFD was more prevalent in females and older adults. Unilateral PCFD limbs demonstrated significantly higher FPI scores (flatter feet) than contralateral healthy limbs. Both sexes demonstrated significant pain reduction following CFOs use, with females reporting higher pain at baseline and follow‐up. Mean pain decreased from 6.52 ± 2.56 at assessment to 3.69 ± 2.52 at follow‐up (*p* < 0.0001). Eighty percent of patients reported symptoms improvement, and 56% wore orthoses for more than 6 hours per day. Comfort was positively associated with both wear time and magnitude of pain reduction.

**Conclusions:**

CFOs were associated with meaningful pain reduction, improved function, and high patient‐reported benefit in individuals with PCFD. These findings support CFOs as a cost‐effective conservative intervention and provide essential data to inform the design of a future randomised controlled trial evaluating their clinical efficacy.

**Abbreviations**
CFOs: Custom foot orthoses
FHL: Functional hallux limitus
FPI: Foot Posture Index
MPJ: Metatarsophalangeal joint
PCFD: Progressive collapsing foot deformity
RCT: Randomised controlled trial

## 1 Background

Musculoskeletal disorders of the foot and ankle are highly prevalent in both the general population and athletic cohorts [1, 2]. Among these, progressive collapsing foot deformity (PCFD) represents a clinically significant condition [3]. Historically, this disorder has been described under various terminologies, including posterior tibialis tendon dysfunction and adult‐acquired flatfoot deformity [4, 5, 6]. Recently, a consensus group composed of orthopaedic surgeons recommended the adoption of the term PCFD, as it could more accurately reflect contemporary understanding of the disorder and promote greater standardisation in clinical characterisation and reporting [3]. PCFD is a chronic and painful musculoskeletal condition that predominantly affects middle‐aged women and older adults, with the highest incidence observed in individuals aged 61–75 years [7, 8, 9]. Restricted physical and social participation [10], along with impaired balance, mobility, and reduced overall strength and endurance [11], collectively lead to diminished foot function, decreased quality of life, and poorer mental health outcomes [12, 13] in affected individuals. Clinically, individuals with PCFD present with abnormal foot and ankle alignment, altered joint moments, and compensatory movement patterns that extend to proximal joints during walking and other dynamic tasks [11, 14, 15, 16, 17, 18, 19].

In the United Kingdom, the James Lind Alliance, whose mission is to establish national health research priorities, identified among its top three research priorities in foot and ankle health the need to determine the most effective treatment for PCFD [20]. This prioritisation underscores the urgency of developing evidence‐based therapeutic strategies to improve the clinical management of this condition. Early‐stage management of PCFD with non‐surgical approaches, such as custom foot orthoses (CFOs), seem to reduce pain and alleviate mechanical stress on the injured joints and soft tissue [21, 22, 23, 24, 25]. CFOs support the medial longitudinal arch, improve hindfoot/forefoot alignment, and enhance ankle plantarflexion in individuals with PCFD [21, 22, 23, 26]. Current evidence further suggests that CFOs may help restore functional capacity and relieve pain in this population [27, 28]. However, no randomised controlled trial (RCT) have isolated CFOs as the sole intervention in an experimental group, making it difficult to determine their true clinical efficacy. To date, evidence regarding optimal treatment strategies for PCFD remains limited and heterogeneous, leaving clinicians uncertain about the most appropriate management approach for their patients [29, 30, 31]. The lack of robust, evidence‐based clinical guidelines impedes the establishment of a clinical consensus, and the development of management protocols grounded in best practice. In this context, conducting a rigorous clinical trial on CFOs is essential to identify the most effective therapeutic strategies. Such research would generate high‐quality evidence to guide clinical decision‐making, establish clear recommendations, and ultimately improve the management of PCFD by reducing clinical uncertainty and optimising patient outcomes. Given the complexity and functional limitations associated with PCFD, custom devices are generally recommended over prefabricated orthoses, although this recommendation is still largely supported by anecdotal evidence [32]. Nevertheless, to inform the design of a robust RCT, comprehensive clinical data on the patient profile, including real‐world clinical observations beyond demographic data, and the effectiveness of CFOs in improving function and alleviating pain are required. Thus, the objectives of this study were to characterise the profile of patients with PCFD seeking treatment in a footwear and orthotic clinic (and confirm the biological sex differences previously reported in the literature), determine if CFOs are effective in reducing pain and improving function in PCFD patients, and determine the variables which correlate with improved pain and function after wearing FOs. It was hypothesised that 1) there would be higher prevalence of females and older adults experiencing PCFD and seeking footwear and orthotic treatment (compared to males and younger adults), 2) patients with higher self‐reported pain levels at assessment will experience the greatest reductions in pain at follow‐up, and 3) CFOs will significantly reduce pain and improve function in patients suffering from PCFD.

## 2 Methods

A retrospective analysis was conducted on clinical data collected from patient assessments with a Canadian Certified Pedorthist between January 1, 2016 and May 31, 2025. Certified pedorthists are specialists in CFOs, lower limb anatomy and gait biomechanics. All clinicians complete a 4‐year undergraduate degree (most commonly in Kinesiology), followed by a pedorthic diploma that includes pedorthic‐specific coursework and 1500+ hours of clinical training and CFOs fabrication. The dataset comprised 130,365 clinical appointments from a private foot care provider (Kintec Footlabs Inc.) with clinic locations in British Columbia and Ontario, Canada. Prior to attending any appointment, all patients provide informed consent to the use of de‐identified clinical data for research purposes. Patient visits were classified into two groups: (1) the PCFD group, including unilateral (left or right) and bilateral presentations, and (2) an asymptomatic control group, consisting of individuals who sought clinical care without reporting pain or symptoms in the feet or lower limbs. Paediatric patients (< 18 years) were removed from both datasets. PCFD assessment data were obtained from individuals who were either self‐referred or referred by a primary healthcare professional (e.g., physician, specialist) to one of 25 private foot care clinics (Kintec Footlabs Inc.). PCFD was verified through clinical assessment encompassing history taking, evaluation of presenting condition, palpation, range of motion and functional testing, and weightbearing examination. Given that our clinical data dates to 2016, the criteria for PCFD corresponding to Johnson and Strom's stage I and II classifications were applied. These standards were commonly used in the literature prior to 2020 and aligns with the prevailing inclusion‐criteria conventions across studies during that period. Clinically, PCFD was confirmed if the following clinical features were present: pain along the posterior tibial tendon during weightbearing, pain on palpation of the tendon or surrounding area, and mild to severe weakness of the tibialis posterior tendon. Tendon inflammation may or may not have been observed. The absence of other conditions, including deltoid ligament rupture, and arterial, vascular, or neuromuscular disorders, was also confirmed. Conversely, the control group consisted of individuals seeking CFOs in the absence of pain or identifiable biomechanical abnormalities. These patients typically pursued orthoses for reasons such as enhanced comfort, performance optimisation, improved balance control, or proprioceptive and sensory benefits.

### 2.1 The Patient Journey

Each patient underwent a comprehensive clinical assessment completed by a Canadian Certified Pedorthist. The assessment included a detailed medical history, characterisation of the primary symptoms affecting the plantar surface of the foot and lower leg, specific location and description of pain, as well as information regarding activities of daily living and typical footwear use. Pain severity was recorded using a numerical rating scale ranging from 0 (no pain) to 10 (worst pain imaginable), applied to the symptomatic limb or bilaterally in cases of PCFD affecting both limbs.

Foot posture was evaluated using the Foot Posture Index (FPI) [33], which classifies static foot alignment along a spectrum from highly cavus (FPI scores of −5 to −12) to highly planus (FPI scores of +10 to +12). Gait analysis is routinely performed in pedorthic practice to help patients understand how their presenting condition relates to their locomotor biomechanics. Although these results are not reported in our results (as they do not relate to the objectives of the study), gait analysis was performed using video recordings (Coach's Eye, TechSmith Corp., Apple iPad, generations 7–9) of patients walking with and without footwear, either in a hallway or on a treadmill. Gait was characterised by kinematic observations at initial contact, midstance, and toe‐off, and all observations were verified through slow‐motion review of the recordings on the iPad. Following gait analysis, non‐weightbearing and weightbearing range of motion was assessed at the first metatarsophalangeal joint (MPJ), along with double‐ and single‐heel raise tests. During the weightbearing hallux dorsiflexion test, also known as the Hubscher manoeuvre, patients were instructed to stand in quiet static stance with equal weight distributed across both feet. The pedorthist passively dorsiflexed the hallux, one foot at a time, and observed the range of motion at the MPJ along with any elevation of the medial longitudinal arch. A positive test is indicated by limited or absent MPJ dorsiflexion and a lack of arch elevation, reflecting impaired engagement of the windlass mechanism. This diminished windlass response is consistent with the biomechanical consequences of PCFD, wherein reduced dynamic support of the medial longitudinal arch increases reliance on passive structures such as the plantar fascia, contributing to a compromised ability to elevate the arch during hallux dorsiflexion. For the heel raise tests, patients were asked to lift their heels off the ground, using both feet simultaneously for double heel raises and one foot at a time for single heel raises, to assess the strength and function of tibialis posterior muscle. This test is typically completed 1–3 times and a positive result is characterised by the absence of calcaneal inversion, reflecting impaired tibialis posterior activation.

### 2.2 Standardised Treatment

After completing the physical examination, the pedorthist developed and explained an individualised treatment plan for each patient. As part of standard clinical practice, footwear recommendations were tailored to the patient's usual footwear habits, occupational demands, and daily activity levels. In most cases, high‐quality footwear was advised to support the patient's foot posture and activity needs. However, the pedorthist also considers variations when occupational requirements differ, when more supportive footwear is necessary, for example, when the patient experiences the greatest pain, or when certain footwear features are beneficial to the patient (i.e., laces vs. Velcro closures, or harder midsole material for added stability and/or dynamic stability). Three‐dimensional foot morphology was captured using a volumetric laser scanner (Occipital, Stockholm, Sweden), while the patient was seated. During scanning, the pedorthist applied a plantarflexory force to the 4‐5th metatarsophalangeal heads to align the forefoot plantigrade with the rearfoot. Scans were then sent to Kiwi Orthotic Services (Surrey, British Columbia, Canada) for CFOs manufacturing.

In approximately 80% of cases, the orthotic shell was fabricated through computer‐assisted‐design and manufacturing system directly milling the polypropylene shell based on a 3‐dimensional processing sequence that closely matches the traditional analogue process; namely the foot scans are balanced to align the forefoot to rearfoot in the frontal plane, appropriate ‘backfill’ or dressing is applied to the foot model to correspond with tissue expansion and shell thickness is determined based off of patient's weight. The shell thickness corresponded with a support rating determined by the assessing pedorthist and were distributed as follows: 74.6% semi‐rigid (shell thickness range = 44 kg = 2.7 mm–119 kg = 4.5 mm), 27% semi‐flex (shell thickness range = 44 kg =2.4 mm–119 kg = 3.9 mm), and 5% rigid (shell thickness range = 44 kg = 3.2 mm–119 kg = 4.9 mm). No shells were categorised as flexible (shell thickness range = 44 kg = 2 mm–119 kg = 3.3 mm) and most top covers were 3 mm neoprene. Cast processing software (Orthomodel, AutoDesk, US; FitFoot360, UK) was used to apply additional rearfoot and/or forefoot posting, in the form of varus or valgus posting, as prescribed at the discretion of the pedorthist.

CFOs were dispensed approximately 2 weeks after the initial assessment. Devices were fit to patients' footwear either by replacing the existing prefabricated sock liner or, in the case of lower‐profile designs, by overlaying the orthosis on the insole. Patients were instructed to adhere to a progressive wear schedule, increasing orthosis use by 1 hour per day until full‐time wear was achieved. If adequate comfort had not been attained within 3 weeks, patients were advised to return for an adjustment consultation. Three weeks following the patient's CFOs fitting appointment, an automated follow‐up email was sent to collect information on patient's global rating of change, pain status, functional ability, and average daily wear time with their CFOs.

### 2.3 Statistical Analysis

All data were assessed for normality using the Kolmogorov–Smirnov test. To describe the clinical profile of patients with PCFD, demographic variables were summarised as means and standard deviations. Associations between self‐reported pain at assessment and demographic measures (age, body mass, and FPI) were examined using Spearman's rho correlation coefficients (SPSS Statistics, Version 30.0.0.0 (172), IBM Corp.). Dependent *t*‐tests were used to analyse the differences between FPI scores of the PCFD patients' healthy compared to affected feet, and in unilateral cases, independent *t*‐tests were used to compare the affected foot to right feet of healthy controls.

To determine the effectiveness of FOs in reducing pain and improving function in PCFD patients, pain scores between assessment and follow up in the PCFD group were analysed using a dependent *t*‐test. Previous literature suggests a change in pain score of 0.895–1.48 out of 10 as a minimal clinically importance difference for numerical rating scales [34]. A two‐factor mixed methods ANOVA was used to evaluate pain scores between biological sexes at assessment compared to follow up. Descriptive statistics summarised means and standard deviations on PCFD patient's perceptual responses to symptom recovery (global rating of pain and physical function).

Lastly, to determine the variables which correlate with improved pain and function after wearing FOs, the relationships between perceived comfort and orthoses wear time, pain at assessment and change in pain scores, and perceived comfort and change in pain scores were evaluated using Spearman's correlation coefficient *r*_s_. Assumptions for Spearman's correlation were satisfied, and equality of variances was tested using the Folded F test. Correlational results were interpreted according to conventional thresholds: strong (*r*_s_ ≥ 0.70), moderate (0.30 ≤ *r*_s_ < 0.70), and weak (*r*_s_ < 0.30). Statistical significance was set at *α* = 0.05.

## 3 Results

The control group consisted of 12,621 encounters from asymptomatic patient visits to the pedorthists, all reporting a pain score of 0.0 ± 0.0. The PCFD group included 669 patients encounters, comprising 283 patients with left‐sided symptoms, 258 with right‐sided symptoms, and 128 patients reporting bilateral pain. Eleven PCFD cases were excluded from the analysis due to incomplete chart documentation regarding the affected limb.

### 3.1 The Clinical Profile of Patients With PCFD

**FIGURE 1:**
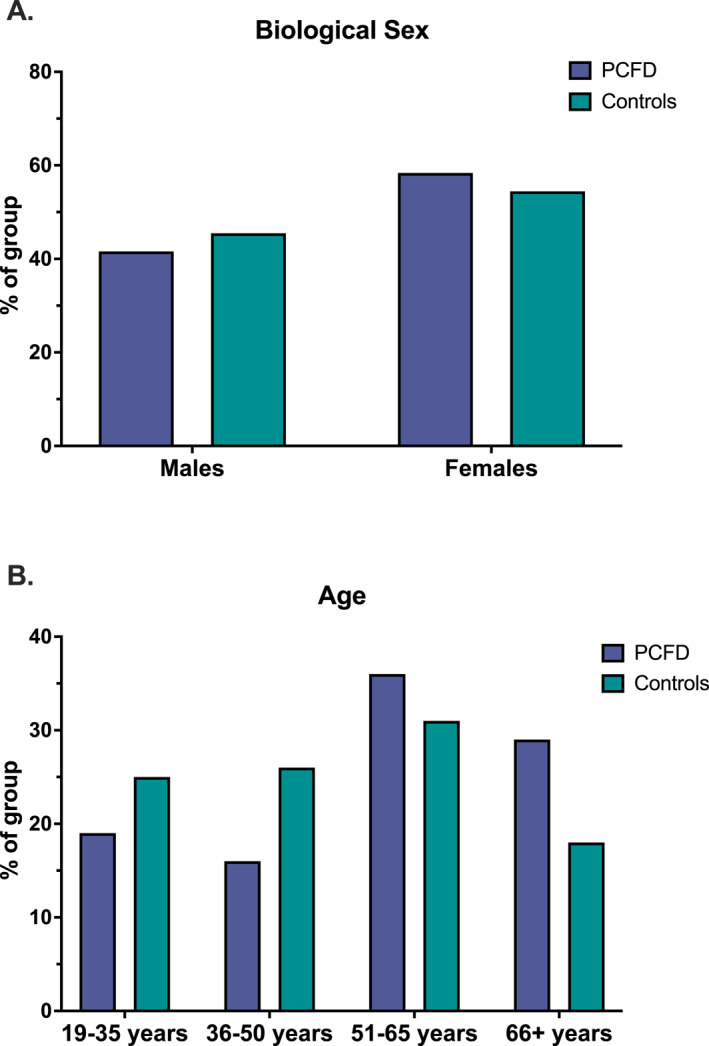
(A) Male and female distribution across the control and PCFD groups and (B) Age distribution across the control and PCFD groups.

Demographic findings indicate that PCFD occurs in both males and females; however, patients with PCFD were slightly older than healthy controls (mean age 54.6 vs. 49.4 years) and showed a greater prevalence among females aged 51–65 years (Figure 1). Mean BMI was also higher in the PCFD group (28.1) compared with controls (26.2). In unilateral PFCD patients, the FPI scores of the affected limb (8.0 ± 3.9) were significantly higher (t (290) = 9.02, *p* < 0.0001, *d* = 2.86), suggesting a flatter foot type, compared to the healthy limb (6.5 ± 3.7). This observation stands in contrast to the normative FPI values observed in the control group (left: 5.6 ± 4.0, right: 5.4 ± 4.0). Furthermore, the FPI scores of the affected foot in unilateral PCFD cases were also significantly higher than the right (dominant) feet of the healthy controls (t (290) = 108.80, *p* < 0.0001, *d* = 2.24). Significant but weak correlations were observed between self‐reported pain and body mass (*r*_s_ = 0.11, *p* = 0.008), and self‐reported pain and BMI (*r*_s_ = 0.13, *p* = 0.004) of PCFD patients. Clinical testing further showed higher proportions of positive double and single heel‐raise tests in the PCFD cohort (double: left = 24.0%, right = 32.0%; single: injured side = 50%–57%) compared with controls (double: left = 2.4%, right = 2.3%; single: left = 9.5%, right = 8.2%). No significant between‐group differences were observed in weightbearing hallux dorsiflexion. Complete demographic details are provided in Table 1, results of the functional tests are provided in Table 2, while age and sex distributions are included in Figure 1.

**TABLE 1:** Demographics.

Group	Demographics, FPI and pain		
Controls *n* = 12621	Age (years): 49.4 ± 16.8	FPI:	
		Left: 5.6 ± 4.0	
	Sex: 5682 females	Right: 5.4 ± 4.0	
	6456 males	Worst pain: 0.0 ± 0.0	
	480 unidentified		
	Height (m): 1.72 ± 0.12		
	Weight (kg): 77.5 ± 18.1		
	BMI: 26.2 ± 5.0		
PCFD *n* = 669	Age (years): 54.6 ± 17.0	FPI: Unilateral cases:	Bilateral cases:
	Sex: 379 females	Healthy: 6.5 ± 3.7	Left: 7.8 ± 3.7
	264 males	Injured: 8.0 ± 3.9	Right: 7.6 ± 3.7
	26 unidentified		
	Height (m): 1.69 ± 0.15	Worst pain: 5.5 ± 2.7	
	Weight (kg): 81.1 ± 19.6		
	BMI: 28.1 ± 6.2		

**TABLE 2:** Functional tests.

Group	Test					
	Weightbearing hallux dorsiflexion (% cases)		Double heel raise test (% cases)		Single heel raise test (% cases)	
Controls	Left:		Left:		Left:	
	Rigid: 2.6		Positive: 2.4		Positive: 9.5	
	FHL: 62.1		Normal: 97.6		Normal: 90.5	
	Normal: 35.3					
	Right:		Right:		Right:	
	Rigid: 2.8		Positive: 2.3		Positive: 8.2	
	FHL: 62.1		Normal: 97.7		Normal: 91.8	
	Normal: 35.1					
PCFD	Unilateral cases:		Unilateral cases:		Unilateral cases:	
	Healthy:	Injured:	Healthy:	Injured:	Healthy:	Injured:
	Rigid: 3.0	Rigid: 3.0	Positive: 12.8	Positive: 50.7	Positive: 9.2	Positive: 21.3
	FHL: 57.8	FHL: 64.9	Normal: 87.2	Normal: 49.3	Normal: 90.8	Normal: 68.7
	Normal: 39.1	Normal: 32.1				
	Bilateral cases:		Bilateral cases:		Bilateral cases:	
	Left:	Right:	Left:	Right:	Left:	Right:
	Rigid: 3.7	Rigid: 3.7	Positive: 24.0	Positive: 32.0	Positive: 30.8	Positive: 33.3
	FHL: 59.3	FHL: 55.6	Normal: 76.0	Normal: 66.7	Normal: 69.2	Normal: 66.7
	Normal: 37.0	Normal: 40.7				

Abbreviation: FHL: functional hallux limitus.

### 3.2 The Effectiveness of CFOs on Reducing Pain and Improving Function

**FIGURE 2:**
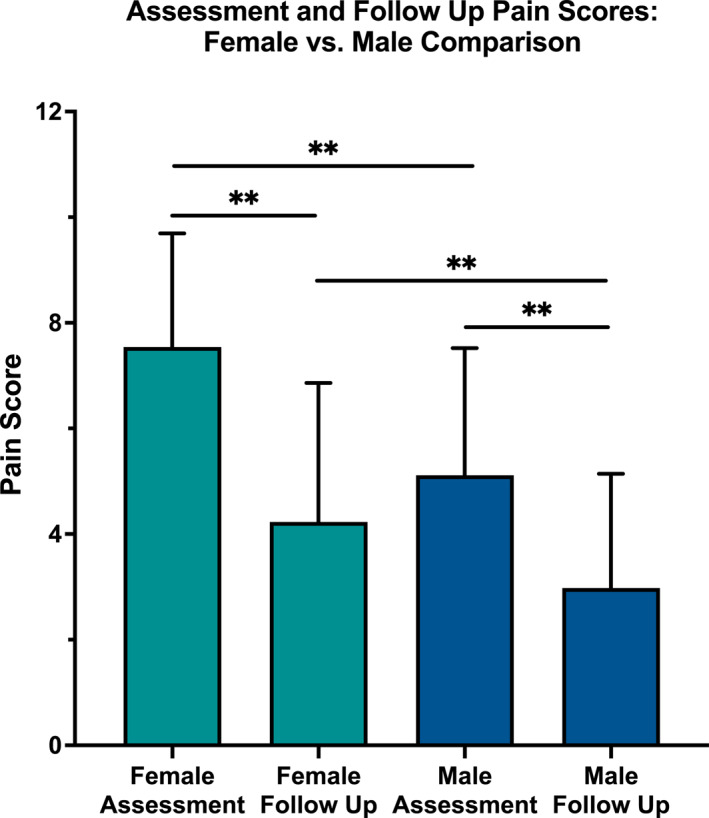
(A) Mean pain score at assessment and at follow‐up in male and female participants with PCFD.

**FIGURE 3:**
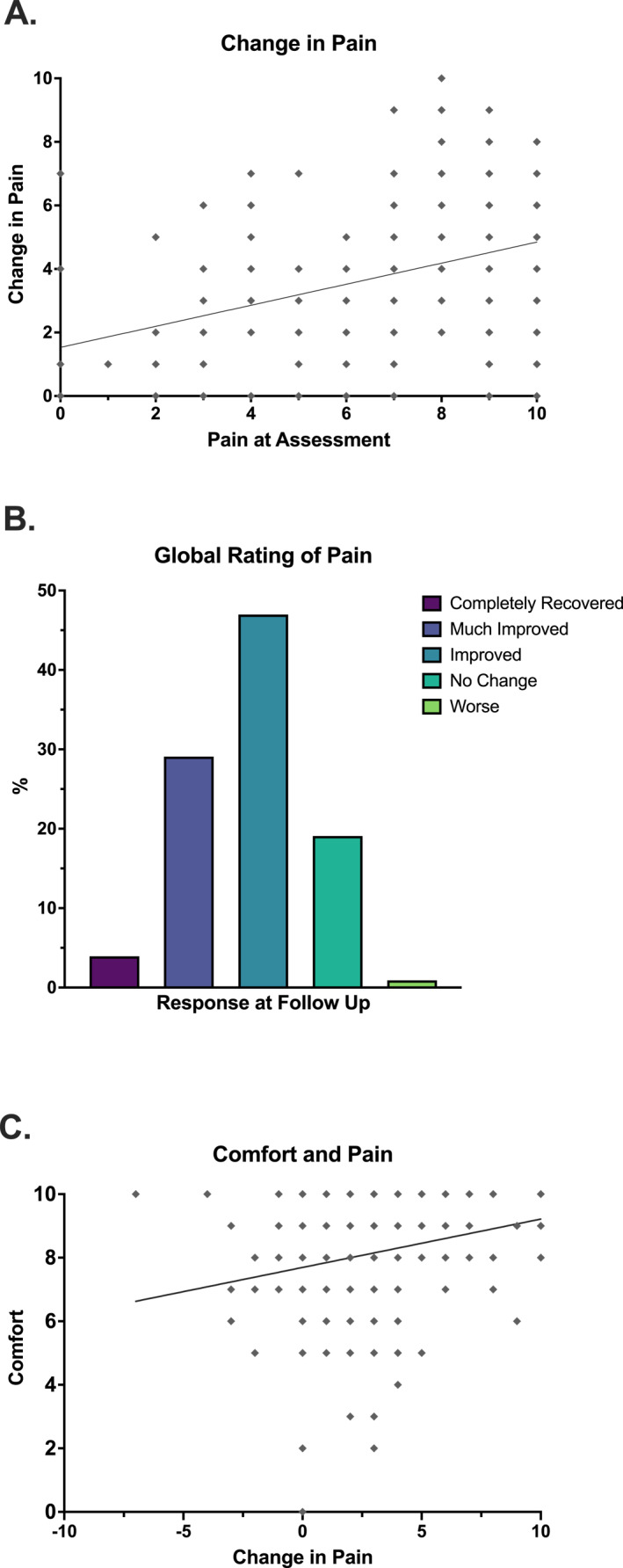
(A) Correlation between pain at assessment and at follow‐up after wearing CFOs, (B) Global rating of pain at follow‐up and (C) Correlation between CFOs comfort and change in pain in patients with PCFD.

Our results suggest that CFOs are an effective treatment modality to reduce pain and improve function in PCFD patients. At follow up (39.7 ± 21.8 days post‐fitting), 56% of PCFD patients reported wearing their CFOs for more than 6 hours/day, while 22% wore them 4–6 hours, and 16% for 1–3 hours daily (5% = couple times/week, 0.5% = couple times/month, 0.5% = never wore orthotics). On average, self‐reported pain at follow‐up (3.7 ± 2.5) was significantly lower than pain at assessment (6.5 ± 2.6) (t (329) = 17.59, *p* < 0.0001, *d* = 2.92), indicating a significant improvement in symptoms among patients with PCFD. A statistically significant interaction (F_3,654_ = 10.05, *p* < 0.0001, *R* ^2^ = 0.344), including a significantly large effect size, was found between male and female pain scores across assessment and follow up (Figure 2). At assessment, females (7.5 ± 2.2) reported significantly higher pain scores than males (5.1 ± 2.4), and this difference persisted at follow up (females: 4.2 ± 2.6, males: 3.0 ± 2.2). For both sexes, pain scores decreased significantly at follow‐up compared to assessment, indicating overall pain reduction. Perceptual responses to symptom recovery were positive in 80% of PCFD patients, with 3.9% reporting completely recovery of symptoms, 29.1% much improved, and 47.0% as improved (Figure 3B). Regarding physical function, 75.5% of patients at follow‐up self‐reported their impairment as none or mild, 19.4% as moderate, and the remaining 5.2% as severe.

### 3.3 Correlations With Improved Pain and Function After Wearing CFOs

Following the use of CFOs, our correlational analysis revealed that longer wear times were positively correlated with comfort (*r*_s_ = 0.25, *p* < 0.0001, *R* ^2^ = 0.06), suggesting that patients who were most comfortable in the CFOs wore the devices for longer periods of time. Pain levels at initial assessment were positively correlated with change in pain (self‐reported pain at follow‐up from assessment) (*r*_s_ = 0.34, *p* < 0.0001, *R* ^2^ = 0.12) indicating that patients who initially presented with higher pain tended to experience greater symptom reduction with orthoses wear (Figure 3A). Additionally, change in pain scores were significantly associated (*r*_s_ = 0.24, *p* < 0.0001, *R* ^2^ = 0.06) with perceived comfort in CFOs, suggesting that patients who rated their orthoses are more comfortable also report greater improvements in pain (Figure 3C).

## 4 Discussion

This is the first Canadian study to report the epidemiology of PCFD in patients seeking care administered through CFOs, filling a critical gap in the literature and offering a foundation for clinical practice guidelines and to inform the design of a comprehensive RCT. Based on the authors' collective clinical experience treating PCFD in diverse practice settings, the characteristics of this cohort closely mirror those of patients with PCFD seeking foot orthoses care across Canada. While additional multicentre research is warranted, the trends observed in this study are highly representative of the population clinicians encounter nationwide. Consistent with epidemiology data from Sweden and the United States, our demographic results indicate that females aged 51 years and older experience a higher prevalence of PCFD compared to both males and age‐matched controls (Figure 1) [7, 8, 9]. In unilateral cases, the affected side typically presents with a flatter foot compared to the contralateral limb and is accompanied by a positive double and single heel raises tests, suggesting impaired medial ankle soft tissue and/or posterior tibial tendon function [35]. Although not directly measurable in this study, it also worth noting that individuals with PCFD likely exhibit anatomical variations of the medial malleolus, such as changes in retromalleolar groove angle and width, which predispose these feet to a higher risk of developing this clinical pathology [36].

On average, 39.7 ± 21.8 days after the dispensing of CFOs, pain and function significantly improved in PCFD patients. Notably, 80% of patients reported symptom improvement, self‐assessed as completely recovered, much improved or improved, suggesting that CFOs are an effective conservative intervention for managing pain and enhancing functional outcomes in this population. Conservative management with CFOs not only provides meaningful clinical benefits but is also a highly cost‐effective option, enhancing quality of life by reducing pain and maintaining mobility. This stands in contrast to surgical interventions, which carry greater risk and financial burden [37]. Freedom from pain is particularly important for middle‐aged and older adults, the demographic most affected by PCFD, as it not only enhances overall well‐being and quality of life, but also enables the resumption of physical activity, which is essential for maintaining independence, cardiovascular health, and mental wellness [38].

Previous research has established the biomechanical benefits CFOs for patients with PCFD, demonstrating reductions in ankle eversion angles and inversion moments, decreased midfoot dorsiflexion, and improved support of the medial longitudinal arch during gait [21, 22, 26]. While this study did not measure biomechanical outcomes, it is reasonable to infer that the significant self‐reported pain improvements observed are likely due to the approximate 40‐day use of CFOs promoting these biomechanical corrections. By offloading stress on injured tissues and promoting conditions for healing, CFOs enable symptoms to subside over time. Notably, patients who reported greater comfort while wearing CFOs experienced larger reductions in pain scores (Figure 3C), suggesting that comfort not only enhances compliance but also ensures sustained biomechanical support, further contributing to symptom relief and functional recovery.

This study was driven by the absence of robust evidence‐based clinical guidelines for PCFD management, with the ultimate goal of using these clinical practice insights to inform the design of a future RCT. Our findings highlight two critical considerations for such a trial: females consistently reported higher pain scores at both baseline and follow‐up, and initial pain levels were strongly correlated with outcomes over time. These patterns underscore the need to account for sex differences and baseline pain severity in trial design to ensure accurate stratification, meaningful comparisons, and optimised treatment strategies for PCFD patients.

This study has several limitations that should be acknowledged. First, its retrospective design restricts the ability to establish causal relationships and may introduce selection bias. Second, there was heterogeneity in the CFOs dispensed across participating clinics, which could have influenced treatment outcomes and reduced the consistency of intervention effects. Finally, potential placebo effects were not considered, which may have contributed to observed improvements in pain or function independent of the orthotic intervention.

## 5 Conclusions

These findings provide preliminary evidence supporting the role of CFOs in managing pain and improving function in individuals with PCFD. However, given the retrospective nature of the study and variability in orthotic prescriptions, further research is warranted. The next step is to undertake a robust RCT specifically designed to evaluate the effects of CFOs on pain and functional outcomes in this population. Such a trial would help clarify the true efficacy of orthotic interventions and inform evidence‐based clinical practice.

## Author Contributions

**Kelly Robb:** conceptualization, data curation, formal analysis, methodology, writing – original draft, writing – review and editing. **Michael Ryan:** writing – review and editing. **Gabriel Moisan:** conceptualization, methodology, writing – original draft, writing – review and editing.

## Funding

Gabriel Moisan received funding from the Fonds de Recherche du Québec‐ Santé (FRQ‐S) (#347697; https://doi.org/10.69777/347697).

## Ethics Statement

The authors have nothing to report.

## Consent

Informed consent was provided by each patient to the use of de‐identified clinical data for research purposes.

## Conflicts of Interest

Kelly Robb serves as the Director of Product and Innovation at Kintec, and Michael Ryan is the Vice President of Brand and Innovation at Kintec Footlabs Inc. Both Kelly and Michael maintain professional relationships with the pedorthists conducting the assessments for this study; however, they had no involvement in the assessment outcomes or in the selection of orthotic manufacturing for these patients.

## Data Availability

The datasets used and/or analysed during the current study are available from the corresponding author on reasonable request.
